# Impact of Smoking, Steroid Use and Immunosuppression on Anti-dsDNA Antibodies in Systemic Lupus Erythematosus

**DOI:** 10.3390/ijms26178705

**Published:** 2025-09-06

**Authors:** Richard Borrelli, Stefania Nicola, Federica Corradi, Luca Lo Sardo, Iuliana Badiu, Anna Quinternetto, Ilaria Vitali, Simone Negrini, Luisa Brussino

**Affiliations:** Allergy and Immunology Unit, Department of Medical Science, University of Turin, Mauriziano Hospital, 10128 Turin, Italy; stefania.nicola@unito.it (S.N.); fcorradi@mauriziano.it (F.C.); llosardo@mauriziano.it (L.L.S.); ibadiu@mauriziano.it (I.B.); annaquinternetto@gmail.com (A.Q.); ilariavitali1@gmail.com (I.V.); simone.negrini@unito.it (S.N.); luisa.brussino@unito.it (L.B.)

**Keywords:** lupus, systemic lupus erythematosus, SLE, anti-dsDNA, smoke, therapy

## Abstract

Systemic lupus erythematosus (SLE) is a chronic autoimmune disease involving multiple organs and the production of anti-double-stranded DNA (anti-dsDNA) antibodies. This study evaluated the associations between anti-dsDNA levels and smoking, oral corticosteroid (OCS) use, and immunosuppressive therapy in SLE patients. A retrospective monocentric analysis was performed on 119 SLE patients. Data on smoking history, OCS dosage, and immunosuppressive treatments were collected. Anti-dsDNA levels were assessed using fluorescent enzyme immunoassays and confirmed via indirect immunofluorescence. Smoking was significantly associated with higher anti-dsDNA levels (Spearman’s ρ = 0.292, *p* = 0.0014). Logistic regression identified pack-years (PPY) as a predictor of high anti-dsDNA levels (≥75 U/mL), with a 50% probability at 12.51 PPY and a 75% probability at 17.65 PPY (*p* < 0.001). OCSs were used by 58.82% of patients, with a median prednisone-equivalent dose of 5.0 mg; higher OCS doses correlated weakly but significantly with anti-dsDNA levels (R^2^ = 0.066, *p* < 0.001). Anti-dsDNA levels differed across treatments (*p* = 0.027): MTX vs. AZA/MMF, and belimumab vs. AZA/MTX. Smoking, OCS use, and immunosuppressants influence anti-dsDNA levels. Belimumab showed greater reduction compared to conventional therapies. Personalized treatment strategies are needed, considering the effects of smoking and cortico-steroids.

## 1. Introduction

### Background

Systemic lupus erythematosus (SLE) is an autoimmune disorder characterized by an involvement of numerous organs, with a complex interaction of immune cells, factors, and pathways, leading to diverse clinical presentations [1].

Among the various autoantibodies, anti-double-stranded DNA antibodies (anti-dsDNA) play an important role in both the pathogenesis and the diagnosis of SLE; its IgG form was first identified in the serum of SLE patients in 1957 by four different groups [2,3], whilst its IgM and IgA counterparts required a few more decades to be properly assessed [3].

From a pathogenic perspective, self-dsDNA is typically restricted to the nucleus and mitochondria, while specific enzymatic pathways ensure its degradation upon release. However, defects in these clearance mechanisms—such as impaired removal of apoptotic debris or neutrophil extracellular traps (NETs)—can lead to the accumulation of self-DNA, contributing to immune activation. This process is fundamental in triggering autoimmunity, as the presence of self-dsDNA in immunologically privileged sites can disrupt tolerance and drive the production of autoantibodies by autoreactive B cells [4].

A central pathway in SLE pathogenesis involves type I interferon (IFN-I) production by plasmacytoid dendritic cells (pDCs) upon interaction with intracellular DNA sensors. IFN-I plays a pivotal role in disease progression by promoting dendritic cell maturation, activating autoreactive T and B cells, and amplifying inflammatory responses. The formation of immune complexes (ICs) composed of anti-dsDNA antibodies and self-DNA further exacerbates inflammation by facilitating the uptake of nucleic acids into antigen-presenting cells, thereby sustaining the IFN-I response and contributing to tissue damage [5,6].

Dysregulated cell death mechanisms, including apoptosis, necrosis, and NETosis, serve as major sources of self-dsDNA in SLE. In healthy individuals, apoptotic cells are rapidly cleared by phagocytes in a non-inflammatory manner. However, inefficient clearance leads to secondary necrosis and the release of immunogenic nuclear material, including modified self-dsDNA. Similarly, defective NET clearance—often due to impaired DNase I activity—results in the persistence of self-DNA in circulation, further fueling the autoimmune response [7].

In our center, anti-dsDNA antibodies were assessed using fluorescent enzyme immunoassays (FEIAs) and confirmed via *Crithidia luciliae* indirect immunofluorescence.

Regardless of the testing procedure, the 2019 ACR/EULAR classification criteria for SLE only require the presence of any positive assay for anti-dsDNA measured with a ≥ 90% specificity [8]. Although, to date, a specific method is not suggested, the recommendations have evolved over time; in fact, in 1997, an anti-dsDNA with abnormal titers was considered positive [9], while in 2012, an anti-dsDNA test was required to be above the reference range or twice above if measured by an enzyme-linked immunosorbent assay [10].

The pathogenic significance of anti-dsDNA is yet to be thoroughly understood; however, its clinical significance in patients with SLE has been proven time and time again [6,11].

In fact, they have been included as one of the classification criteria of SLE since 1982 [12]; their presence has often been associated with disease activity, particularly in renal and cutaneous involvement, and can be influenced by various factors, including genetic predisposition, environmental triggers, and treatment regimens [13].

Given the multifactorial nature of SLE pathogenesis, the production, specificity, and pathogenic behavior of anti-dsDNA antibodies can be influenced by various environmental and therapeutic factors. Several studies indicate that factors such as smoking and the use of certain therapies (e.g., corticosteroids and immunosuppressive agents) may influence the immune response in SLE patients, potentially affecting the production of anti-dsDNA antibodies. Smoking, in particular, is recognized for its immunomodulatory effects and has been linked to the worsening of autoimmune diseases [14]. Furthermore, it has been associated with a reduced efficacy of treatments like belimumab and hydroxycloroquine, particularly in mucocutaneous forms of SLE [15,16,17].

Similarly, the use of oral corticosteroids (OCSs) in managing disease flares could influence antibody production and immune regulation [18].

Considering the multifactorial nature of anti-dsDNA antibody production in SLE, it is crucial to explore these potential determinants in greater detail.

The present study aims to quantify the relationship between modifiable (e.g., smoking) and treatment-related (e.g., corticosteroids and immunosuppressants) factors with anti-dsDNA levels in a stable outpatient SLE population. While previous reports have highlighted the potential impact of smoking or belimumab on serological profiles, our analysis provides probabilistic thresholds (e.g., pack-years and antibody levels) that may assist in identifying persistent subclinical immune activation. This may inform clinical decisions regarding smoking cessation and the use of B-cell-targeted therapies.

To our knowledge, this is the first study to provide probabilistic cut-offs of smoking exposure associated with anti-dsDNA positivity in a stable SLE outpatient cohort. By extending prior evidence into a real-world, low-disease-activity setting, our work underscores the clinical relevance of monitoring modifiable factors such as smoking and tailoring therapy accordingly.

## 2. Results

**Figure 1:**
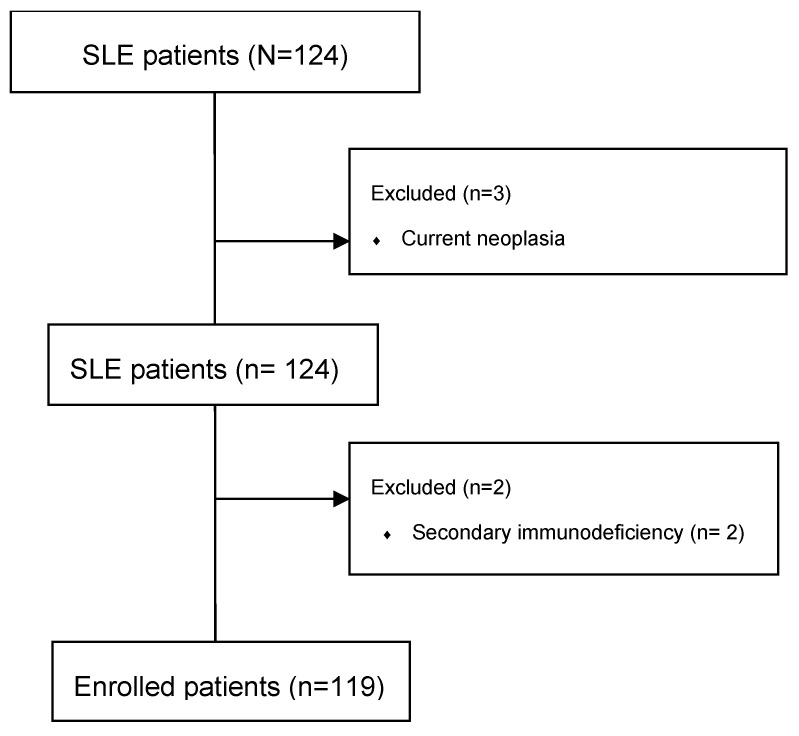
Study flowchart.

One-hundred and nineteen patients were enrolled in the study (107 F, 12 M, F:M 8.92:1). The study flowchart can be seen in Figure 1, whilst the cohort characteristics (demographics, comorbidities and therapies) are presented in Table 1, Table 2, Table 3 and Table 4.

As indicated in Table 2 cardiovascular disease refers to conditions such as blood hypertension, coronary artery disease, and heart failure. Endocrine/metabolic comprises diabetes, thyroid disorders, and metabolic syndromes. Gastrointestinal disease covers liver disorders, inflammatory bowel disease, and other digestive system conditions. Hematology includes anemia and coagulation disorders. Neurologic/psychiatric encompasses epilepsy, dementia, depression, and other neuropsychiatric conditions. Musculoskeletal refers to arthritis, osteoporosis, and other disorders of the musculoskeletal system. Others/unclassified includes comorbidities not falling into the predefined categories or not otherwise specified.

To assess the impact of smoking on anti-dsDNA levels, data on smoking history, duration, and quantity were collected from patients; the data are shown in Table 5. A chi-square test and post hoc pairwise comparisons with Bonferroni correction were performed.

Both logistic regression and linear regression analyses were performed; in the logistic regression model, the threshold for high anti-dsDNA levels was set at 75 U/mL. The number of critical pack-years (PPYs) associated with a 50% probability of exceeding this threshold was 12.51 PPYs, while the 75% probability threshold was 17.65 PPY (*p* < 0.001).

**Figure 2:**
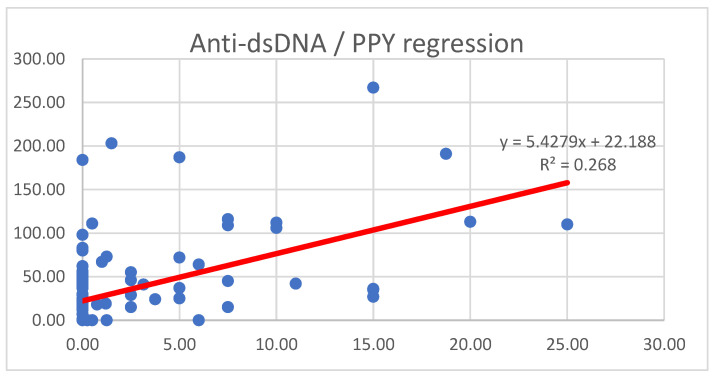
Linear regression between anti-dsDNA levels and PPY. PPY = pack-years; anti-dsDNA: anti-double-stranded DNA antibodies. Y-axis: levels of anti ds-DNA antibodies (UI/mL). X-axis: number of PPYs. β = 5.43, 95% CI: 3.79 to 7.06; *p* < 0.001.

The linear regression analysis revealed a significant positive association between PPYs and anti-dsDNA levels, as illustrated in Figure 2.

Moreover, a logistic regression model evaluating the different smoking status showed that current smoking was significantly associated with increased odds of anti-dsDNA positivity (OR 11.8, 95% CI 3.76–37.4, *p* < 0.001), whereas former smoking was not significantly associated with an increased seropositivity (*p* = 0.25).

The slope of 5.43 (*p* < 0.001) suggests that, in smokers, for each additional pack-year, anti-dsDNA levels increase on average by 5.43 U/mL. The model demonstrated a moderate explanatory power with an R^2^ of 0.268, indicating that approximately 26.8% of the variance in anti-dsDNA levels could be attributed to smoking exposure.

Regarding oral corticosteroids (OCSs), seventy patients (58.82%) were receiving treatment, with a median prednisone-equivalent dosage of 5.0 mg; the interquartile range extended from 1.44 mg (Q25) to 10.0 mg (Q75).

**Figure 3:**
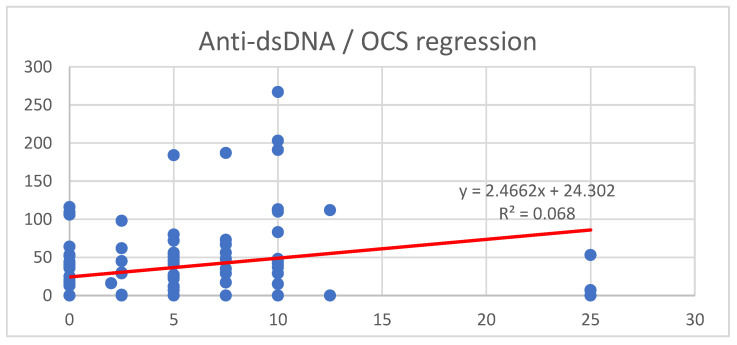
Linear regression between anti-dsDNA levels and OCS. Y-axis: levels of anti-ds-DNA antibodies (UI/mL). X-axis: prednisone equivalent (mg/day). β = 2.47, 95% CI: 0.77 to 4.08; *p* = 0.005.

To assess the relationship between OCS dosage and anti-dsDNA levels, a linear regression analysis was conducted; the results are illustrated in Figure 3.

The model showed a weak positive association between OCS dosage and anti-dsDNA levels (R^2^ = 0.068), indicating that OCS dosage accounted for approximately 6.6% of the variance in anti-dsDNA levels.

Lastly, the effect of therapies on anti-dsDNA levels was evaluated; to assess whether different immunosuppressive therapies had a significant impact on anti-dsDNA levels, a Kruskal–Wallis test was performed, revealing overall differences between treatment groups (*p* = 0.027). Pairwise comparisons identified significant differences between specific therapies; in particular, azathioprine and mycophenolate mofetil (MMF) were associated with significantly different anti-dsDNA levels compared to methotrexate (MTX), while belimumab (BEL) showed significant differences compared to both AZA and MTX. The results are shown in Table 6.

## 3. Discussion

Anti-dsDNA antibodies play a multifaceted role in driving tissue damage in SLE, particularly in lupus nephritis, through a combination of immune complex-mediated inflammation, complement activation, and direct cellular effects.

Recent evidence has highlighted the role of circulating cell-free DNA (cfDNA) not only as a biomarker of disease activity but also as a direct contributor to SLE pathogenesis. cfDNA derives primarily from apoptotic, necrotic, and NETotic cell death and tends to accumulate due to impaired clearance mechanisms in SLE [19]. Once in circulation, cfDNA can form immunogenic complexes with anti-dsDNA antibodies, leading to the formation of pathogenic immune complexes that are prone to deposit in highly vascularized tissues such as the kidneys. These complexes contribute to glomerular inflammation and injury through complement activation and the subsequent recruitment of inflammatory cells. This mechanism is particularly relevant in lupus nephritis, where elevated cfDNA levels have been shown to correlate with disease severity [19,20].

Beyond immune complex-mediated mechanisms, anti-dsDNA antibodies can also exert direct cytotoxic effects. Different studies have demonstrated that a subset of anti-dsDNA antibodies can penetrate living cells—including mesangial, tubular epithelial, and endothelial cells—via mechanisms that are not yet fully elucidated, but may involve Fc receptor-mediated endocytosis or nucleolin-mediated uptake [4]. Once internalized, these antibodies can localize to the nucleus or cytoplasm and bind to DNA or histone complexes, altering gene transcription, promoting mitochondrial dysfunction, and enhancing pro-apoptotic signaling; unlike the previous mechanisms, this intracellular activity contributes to chronic, antibody-driven injury that is not necessarily accompanied by detectable immune complexes.

Another important aspect is the molecular mimicry and cross-reactivity of anti-dsDNA antibodies with glomerular antigens. Some pathogenic clones recognize not only DNA but also non-DNA antigens such as α-actinin, laminin, or components of the glomerular basement membrane [21]. This cross-reactivity facilitates antibody deposition in renal tissues independently of circulating DNA, serving as a “second hit” that localizes inflammation specifically to the kidney.

In order to address the importance of such elements, our study cohort enrolled 119 SLE patients with a female-to-male ratio of 8.92:1, a finding consistent with previous reports indicating a predominant female prevalence in SLE, typically around 9:1 in Western populations [22]. The mean age of 54.62 years and mean disease duration of 25.6 years also align with findings from other studies investigating long-term SLE cohorts [23,24].

In this cohort, 37.82% of patients received azathioprine, 30.25% mycophenolate mofetil, 4.20% methotrexate, and 24.37% belimumab, with 71.42% on hydroxychloroquine. These frequencies are largely consistent with existing data on SLE treatment patterns, where AZA and MMF are among the most commonly used immunosuppressants [25]. The proportion of patients receiving belimumab is comparable to that found in recent studies investigating biologic use in SLE [26].

Notably, belimumab use was particularly frequent in patients concomitantly receiving HCQ (93.1%), aligning with evidence supporting the efficacy of HCQ in enhancing belimumab’s therapeutic effects [27]. The relatively lower percentage of MTX users (4.20%) is consistent with its more limited role in SLE compared to RA, where this DMARD is usually prescribed as first-line therapy [28].

A significant positive correlation was found between pack-years (PPYs) and anti-dsDNA levels, with active smokers displaying the highest prevalence of elevated anti-dsDNA. This finding is supported by previous studies demonstrating that smoking exacerbates autoimmunity and increases anti-dsDNA levels [13,14].

Our stratified analysis of smoking status revealed that current smoking might be significantly associated with anti-dsDNA positivity, whereas former smoking is not. In a logistic regression model adjusted for smoking categories, current smokers showed markedly increased odds of testing positive for anti-dsDNA antibodies compared to never smokers. In contrast, no significant increase in odds was observed among former smokers, implying that the immunomodulatory effects of tobacco exposure may attenuate once smoking is discontinued.

Moreover, ROC analysis further confirmed that the number of PPYs is a predictive marker for elevated anti-dsDNA levels, with a threshold of 12.51 PPYs for a 50% probability and 17.65 PPYs for a 75% probability of having high anti-dsDNA levels. These findings reinforce the hypothesis that smoking contributes to sustained autoantibody production and disease exacerbations, as shown in numerous studies. Moreover, smoking has been previously linked to a reduced response to belimumab and hydroxychloroquine, which may explain the observed associations [15,16].

Concerning OCSs, our study showed a weak but significant association between OCS dosage and anti-dsDNA levels (R^2^ = 0.066, *p* < 0.001). As the nature of the study is retrospective, no causality can be proved; however, higher doses of OCSs are usually prescribed in patients with more severe forms of SLE, which might explain the association observed between increased levels of anti-dsDNA and higher doses of OCSs.

Moreover, while corticosteroids are essential for controlling disease flares, their long-term immunomodulatory effects remain complex. Furthermore, reaching the remission or, if not possible, a low disease activity state (LLDAS), is crucial to beginning a tapering regime in order to reduce the well-documented cumulative dose-dependent adverse effects of corticosteroids (namely osteoporosis, cardiovascular disease, metabolic disturbances), along with cataracts, glaucoma, myopathy, neuropsychiatric complications, and impaired wound healing [29,30]; indeed, in patients with SLE, prolonged corticosteroid use has also been linked to accelerated atherosclerosis, avascular necrosis, and organ damage, contributing to the irreversible accrual of morbidity over time [31]. These dose-dependent toxicities emphasize the importance of minimizing long-term corticosteroid exposure through the early implementation of steroid-sparing agents and optimized disease control strategies.

In our cohort, the SELENA-SLEDAI median score was 2.0 (IQR 0.0–4.0), indicating that the majority of patients had no or mild disease activity at the time of assessment. According to the established cut-offs (no activity = 0; mild = 1–5; moderate = 6–10; high = 11–19; very high ≥ 20), these results suggest a relatively stable population, predominantly in a remission or in a low-disease-activity state. This is consistent with the outpatient and long-term follow-up setting of our study, where aggressive disease flares are less frequently observed.

The low SELENA-SLEDAI values also support the interpretation of anti-dsDNA levels in the context of chronic immune activation rather than acute disease exacerbation. Notably, even in this generally stable population, smoking and OCS use were significantly associated with elevated anti-dsDNA levels, indicating that these factors may influence serological activity independently of clinical disease flares.

Moreover, the different treatments seemed to produce a statistically significant difference in anti-dsDNA levels; post hoc analysis highlighted that AZA and MMF were associated with significantly different anti-dsDNA levels compared to MTX; similarly, belimumab showed significant differences compared to both AZA and MTX.

These results align with previous studies suggesting that belimumab has a greater impact on reducing anti-dsDNA levels compared to conventional immunosuppressants [32]. Notably, MMF did not significantly differ from AZA, consistent with data indicating that both drugs have comparable efficacy in SLE management [33].

Meanwhile, the statistical association between belimumab use and lower anti-dsDNA levels aligns with its mechanism of inhibiting B-cell activating factor (BAFF), a key driver of autoreactive B cell survival.

These findings reinforce the importance of considering smoking habits, corticosteroid use, and therapy selection in managing anti-dsDNA levels in SLE patients. Smoking cessation programs should be emphasized given the strong association between PPYs and sustained autoantibody production. Additionally, belimumab may be particularly beneficial in reducing anti-dsDNA levels, supporting its role in personalized treatment strategies.

Further prospective studies are needed to explore the longitudinal impact of these factors on disease progression, particularly regarding the potential bidirectional effects of OCS on autoantibody regulation and the long-term benefits of belimumab over conventional immunosuppressants.

Beyond confirming known associations, the present study adds novel insights that may have direct clinical implications. By quantifying the relationship between cumulative smoking exposure and anti-dsDNA levels, we provide practical probability thresholds that can assist clinicians in identifying patients at increased risk of persistent serological activity, even in the absence of overt flares. This probabilistic approach, applied to a predominantly stable outpatient cohort, highlights the concept of subclinical immune activation and may support more proactive counseling on smoking cessation. Similarly, the observation that belimumab retains a distinguishable impact on anti-dsDNA reduction compared to conventional immunosuppressants in this “real-world” population strengthens the rationale for its targeted use in patients with persistent serological activity. Together, these findings extend beyond the mere confirmation of prior knowledge by offering quantitative, practice-oriented evidence that could inform both patient education and personalized therapeutic decision-making.

### Limitations

Despite the valuable insights offered by this study, it is important to acknowledge several limitations, which are detailed in the following points:

(1) First, the retrospective study design limits the ability to establish causal relationships between smoking, therapy, and anti-dsDNA levels. While significant associations were identified, prospective longitudinal studies are needed to confirm these findings and further explore the temporal dynamics of these factors in systemic lupus erythematosus (SLE) progression.
(2) Second, smoking status was assessed using cumulative pack-years (PPYs), which provides an estimate of exposure but does not capture potential variations in smoking intensity, duration, or cessation periods. More detailed data, including biomarkers of tobacco exposure, could help refine the analysis.
(3) Third, while anti-dsDNA levels were measured using fluorescent enzyme immunoassays (FEIAs) and confirmed via indirect immunofluorescence (IFA), variability among different laboratory methods may have influenced the results. Future studies could benefit from a standardized multi-platform assessment of autoantibody levels.
(4) Additionally, the cohort was monocentric, potentially limiting the generalizability of the findings to broader SLE populations with different genetic, environmental, or treatment backgrounds. Larger multicenter studies would help validate these observations.
(5) Another key limitation is the temporal sequencing of therapies. Some patients may have received multiple treatments over time, and in some cases, concomitant use of different immunosuppressants could have influenced anti-dsDNA levels. This variability makes it difficult to determine the isolated effect of each therapy. Future studies with longitudinal treatment data and stratified analyses of sequential or overlapping therapies could help clarify these effects.
(6) Finally, while this study focused on anti-dsDNA levels, other disease activity markers (e.g., complement levels, interferon signatures, or specific cytokines) were not included in the analysis; a comprehensive immunological assessment could provide a more detailed understanding of how these factors interact with disease pathophysiology.

Future research should aim to address these limitations by incorporating prospective study designs, more refined exposure assessments, standardized laboratory methodologies, and broader immunological profiling to enhance our understanding of the determinants influencing anti-dsDNA levels in SLE; in particular, prospective studies assessing the evolution of anti-dsDNA titers following smoking cessation may help clarify whether the immunological impact of tobacco exposure is indeed reversible. Similarly, longitudinal evaluations of anti-dsDNA responses to treatment changes—including the introduction, withdrawal, or escalation of immunosuppressive agents and biologics such as belimumab—would provide deeper insights into the immunomodulatory effects of these interventions.

## 4. Materials and Methods

### 4.1. Population and Study Design

This retrospective monocentric analysis comprised outpatients visited from 2017 to 2024 who had been classified as having systemic lupus erythematosus, SLE, as per the 2019 American College of Rheumatology/European Alliance of Associations for Rheumatology (ACR/EULAR) classification criteria.

Medical records were used to verify the presence and level of anti-dsDNA antibodies and oral corticosteroid (OCS) dosage. Smoking habits were expressed as cumulative smoking pack-years (PPYs). Records of immunosuppressive therapies were also collected, including conventional disease-modifying antirheumatic drugs (cDMARDs) such as azathioprine (AZA), mycophenolate mofetil (MMF), and methotrexate (MTX), as well as biologic DMARDs (bDMARDs), specifically belimumab (BEL) and rituximab (RTX). Use of hydroxychloroquine (HCQ) was also documented. For intergroup comparisons, patients were categorized according to their ongoing immunosuppressive regimen at the time of anti-dsDNA measurement.

In cases of sequential therapies, the treatment being administered at the time of blood sampling was considered. For patients undergoing combination therapy, classification was based on the biologic agent, reflecting its more targeted mechanism of action. Patients receiving rituximab were analyzed separately due to the drug’s prolonged immunologic effects and intermittent dosing schedule.

To ensure the integrity of the data, patients with incomplete or insufficient medical documentation were excluded. In addition, we added exclusion criteria for substance abuse, primary or secondary immunodeficiency diagnosis, and current neoplasia.

SELENA-SLEDAI (Safety of Estrogens in Lupus Erythematosus National Assessment—Systemic Lupus Erythematosus Disease Activity Index) is a modified and validated version of the original SLEDAI, designed to quantify disease activity in patients with systemic lupus erythematosus over a 10-day reference period. It incorporates both clinical manifestations and laboratory abnormalities, assigning predefined weights to a set of 24 parameters [34]. These include neurological symptoms such as seizures or psychosis, mucocutaneous involvement, arthritis, serositis, myositis, and renal features including hematuria, pyuria, proteinuria, and the presence of urinary casts. Laboratory measures encompass hypocomplementemia and anti-dsDNA positivity. The score reflects the disease activity at the time of assessment, with total values ranging from 0 to 105. In clinical practice, SELENA-SLEDAI is widely employed to monitor disease evolution, evaluate therapeutic efficacy, and inform treatment adjustments, especially in longitudinal follow-up or clinical trial settings. Based on previously established cut-offs, disease activity is categorized as absent (score = 0), mild (score 1–5), moderate (6–10), high (11–19), and very high (≥20) [34].

Anti-double-stranded DNA antibodies were assessed using fluorescent enzyme immunoassays (FEIAs) by ThermoFisher, Waltham, MA, USA, and IFA on *Crithidia luciliae* (Euroimmun S.r.L, Tokyo, Japan) for positive findings to confirm the result.

### 4.2. Statistical Analysis

All statistical analyses were performed using STATA SE 18.0 (StataCorp, College Station, TX, USA). Data visualization was conducted using Python 3.13 (Matplotlib and Seaborn libraries, version 0.12.2).

Descriptive statistics were used to summarize demographic and clinical characteristics. Continuous variables were tested for normality using the Shapiro–Wilk test. Normally distributed variables were expressed as mean ± standard deviation (SD), while non-normally distributed variables were reported as median and interquartile range (IQR: Q25–Q75).

To assess the association between anti-dsDNA levels and smoking exposure (pack-years, PPY) and oral corticosteroid (OCS) dosage, both Spearman’s correlation coefficient and linear regression analysis were performed. The regression models were tested for linearity assumptions and heteroscedasticity. The predictive ability of PPY on anti-dsDNA levels was further evaluated using receiver operating characteristic (ROC) curve analysis, with thresholds determined for a 50% and 75% probability of high anti-dsDNA levels (≥75 U/mL).

To compare anti-dsDNA levels across different therapeutic groups, a Kruskal–Wallis test was conducted. Post hoc comparisons were performed using Dunn’s test with Bonferroni correction to identify significant differences between specific treatments.

All statistical tests were two-tailed, and a *p*-value < 0.05 was considered statistically significant.

## 5. Conclusions

In conclusion, this study provides important insights into the multifactorial determinants influencing anti-dsDNA antibody levels in SLE. The significant association between smoking, therapy use, and anti-dsDNA levels underscores the importance of considering both environmental and therapeutic factors in managing SLE. Further research is warranted to explore these relationships in more depth and to develop personalized treatment strategies based on these findings.

## Figures and Tables

**Table 1:** Demographics.

	n	Age	SD	Disease Duration (y)	SD
TOT	119	54.62	13.61	25.60	13.54
FEMALE	107	54.3	13.62	25.30	13.62
MALE	12	57.25	13.56	17.98	9.47

**Table 2:** Population comorbidities.

Comorbidity	n (%)
Cardiovascular Disease	32
Endocrine/Metabolic	11
Others/Unclassified	5
Hematology	2
Gastrointestinal Disease	6
Neurologic/Psychiatric	1
Musculoskeletal	1

**Table 3:** Anti-dsDNA levels and Safety of Estrogen in Lupus National Assessment-Systemic Lupus Erythematosus Disease Activity Index (SELENA-SLEDAI).

Parameter	n (%)	Median (Q25; Q75)
Anti-dsDNA positivity	68 (57.14)	44.5 (24.75; 68.25)
SELENA-SLEDAI	119 (100)	2.0 (0.0; 4.0)

Anti-dsDNA: anti-double-stranded DNA antibodies.

**Table 4:** Patients’ therapies.

Therapy	n (%)	Concomitant to BEL (n)	Duration of Therapy, Median (Q25; Q75)
BEL	29 (24.37)	NA	4.10 (2.31; 6.58)
AZA	45 (37.82)	7	11.32 (3.24; 17.44)
MMF	36 (30.25)	4	14.22 (5.25; 17.73)
MTX	5 (4.20)	1	3.21 (1.87; 6.93)
RTX	4 (3.36)	0	2.87 (1.17; 3.81)
HCQ	85 (71.42)	27	24.16 (23.11; 25.42)

BEL: belimumab. AZA: azathioprine. MMF: mycophenolate mofetil. MTX: methotrexate. RTX: rituximab. HCQ: hydroxychloroquine. NA: not applicable. Q25: 25th quartile. Q75: 75th quartile

**Table 5:** Distribution of anti-dsDNA positivity according to smoking status (N, %).

Patients	Anti-dsDNA +	Anti-dsDNA −	Comparison vs. Active Smokers (*p*-Value)
Total, N = 119 (100)	68 (57.14)	51 (42.86)	NA
Active smokers	32 (26.89)	4 (3.36)	NA
Previous smokers	9 (7.56)	7 (5.88)	0.023
Nonsmokers	27 (22.69)	40 (33.61)	<0.001

Anti-dsDNA: anti-double-stranded DNA antibodies. NA: not applicable.

**Table 6:** Comparison of different treatments on Anti-dsDNA levels (Dunn’s test with Bonferroni’s correction).

Therapy No. 1	Therapy No. 2	*p*-Value
AZA	MMF	0.27
AZA	MTX	0.004
AZA	CTX	0.40
MMF	MTX	0.04
MMF	CTX	0.26
BEL	AZA	0.04
BEL	MMF	0.17
BEL	MTX	0.01

BEL: belimumab; AZA: azathioprine; MMF: mycophenolate mofetil; MTX: methotrexate.

## Data Availability

Data will be made available upon request to the corresponding author.

## Abbreviations

The following abbreviations are used in this manuscript: SLE (systemic lupus erythematosus), anti-dsDNA (anti-double-stranded DNA), OCSs (oral corticosteroids), PPYs (pack-years), FEIA (fluorescent enzyme immunoassay), ROC (receiver operating characteristic), AUC (area under the curve), ACR/EULAR (American College of Rheumatology/European Alliance of Associations for Rheumatology), cDMARDs (conventional disease-modifying antirheumatic drugs), bDMARDs (biologic disease-modifying antirheumatic drugs), HCQ (hydroxychloroquine), AZA (azathioprine), MMF (mycophenolate mofetil), BEL (belimumab), SD (standard deviation), and IQR (interquartile range).
